# Response to Article Published in Vol. 5, No. 1. Leiomyosarcoma of the Cephalic Vein

**DOI:** 10.1080/13577140120099227

**Published:** 2001-12

**Authors:** Yener Saglik, Yusuf Yildiz, Fikri Icli


*Sarcoma* (2001) **5**, 217

**LETTER TO THE EDITOR**

### Response to article published in Vol. 5, No. 1. Leiomyosarcoma of the cephalic vein

EDITOR — We have read the article entitled 'Leiomyosarcoma of the cephalic vein' by Jefferson and Dixon,[1] published in *Sarcoma* Vol. 5, No 1.

Although the authors indicate that they have made an extensive literature search, and have found only 34 cases of Leiomyosarcoma of veins localized to extremities, there seems to be some cases missing. We searched through Pubmed and found at least 22 more cases, published between 1965 and 1999, including our manuscript published in International Orthopeadics in 1992[2–18]. There are also two new cases published in 2000.

Leiomyosarcoma of venous origin are rare tumors, of which most originate from inferior vena cava (IVC). Larger veins including renal, hepatic, and superior vena cava are also other common locations. Cases of IVC localization outnumber the remainders by more than 218 cases reported by Mingoli *et al.*[19] in 1996, far more than the number the authors indicate in their introduction.

The internet has made life easier for everyone and we believe that it has also made literature searches faster and more reliable than conventional methods.

Yours sincerely
Yener Saglik, M.D.,
Yusuf Yildiz, M.D. and
Fikri Icli, M.D.

### References

1. Jefferson KP, Dixon JH. Leiomyosarcoma of the cephalic vein. *Sarcoma* 2001; 5: 27–30.
2. Gonzalez FB. Leiomyosarcoma of iliac and femoral veins. *Arch Surg* 1965; 91: 623–4.
3. Larmi TK, Niinimaki T. Leiomyosarcoma of the femoral vein. *J Cardiovasc Surg* 1974; 15: 602–5.
4. Nesbit RR Jr, Rob C. Leiomyosarcoma of a vein: survival for six years. *Arch Surg* 1975; 110: 118–9.
5. Payen L, Caulet T, Diebold MD, Pluot M, Patey M, Bauquel J. Leiomyosarcoma of the wall of the deep femoral vein. Apropos of a case. *arch Anat Cytol Pathol* 1983; 31: 233–6.
6. Joyeux A, Moreau P, Senac JP, Thevenet A. Leiomyosarcoma of the femoral vein. Apropos of a case. *J Mal Vasc* 1984; 9: 221–4.
7. Gandemar M, Dauge MC, Grossin M, Bourgois P, Bocquet L. Peripheral vascular leiomyosarcoma. Aprops of 2 cases. *Ann Pathol* 1987; 7: 56–63.
8. Kaufman JL. Leiomyosarcoma of the popliteal vein (Letter to the editor). *Arch Surg* 1987; 122: 119.
9. van Gulik TM, Taat CW, Slors JF, Bras J, Blank LE, Bakker PJ, Kranhout JG, Brummelkamp WH. Leiomyosarcoma of large and small veins: clinical findings and results of treatment in six patients. *Eur J Surg Oncol* 1991; 17: 125–34.
10. Song KY, Jang YW, Kim MK, Lee GY, Sung RH. Leiomyosarcoma arising in the great saphenous vein. A case reposrt. *J Korean Med Sci* 1991; 6: 372–5.
11. Saglik Y, Icli F, Uluoglu O, Isiklar ZU. Leiomyosarcoma of the great saphenous vein. *Int Orthop (SICOT)* 1992; 16: 185–7.
12. Bradley WD, Fields KB, Delaney MJ. Leiomyosarcoma of the femoral vein in a marathon runner. *J Am Board Fam Pract* 1992; 5: 219–21.
13. Joyce P, Johnson FE, Sundaram M, Janney C. Leiomyosarcoma of the femoral vein. *Can Assoc Radiol J* 1992; 43: 225–6.
14. Fahrig C, Heidrich H Benhrendt C, Heydler B, Stockmann U. Leiomyosarcoma in the area of the pelvic vein. *Vasa* 1993; 22: 264–8.
15. Byard RW, Bourne AJ, Phillips GE, Rice MS, Davey RB. Leiomyosarcoma of the saphenous vein in a child with 12-year follow-up. *J Pediatr Surg* 1993; 28: 271–4.
16. Roy C, Beaujeux R, Mutter D. Leiomyosarcoma of the femoral vein: imaging findings. *Am J Roaentgenol* 1993; 160: 1125–6.
17. Stambuk J, Oddo D, Perez R, Bravo M. Leiomyosarcoma of the saphenous vein. *Rev Med Chil* 1993; 121: 673–6.
18. Reix T, Sevestre H, Sevestri-Petri MA, Szychta P, Pietri J. Primary malignant tumors of thevenous system in the lower extremities. *Ann Vasc Surg* 1998; 12: 589–96.
19. Mingoli A, Cavallaro A, Sapienza P, Di Marzo L, Feldhaus RJ, Cavallari N. International registry of inferior vena cava leiomyosarcoma: analysis of a world series on 218 patients. *Anticancer Res* 1996; 16: 3201–5.

1357–714X print/1369–1643 online/01/040217–01 © 2001 Taylor & Francis Ltd
DOI: 10.1080/13577140120099227
